# Environmental abiotic and biotic factors affecting the distribution and abundance of *Naegleria fowleri*

**DOI:** 10.1093/femsec/fiaa238

**Published:** 2020-11-26

**Authors:** Leigha M Stahl, Julie B Olson

**Affiliations:** Department of Biological Sciences, The University of Alabama, Tuscaloosa, AL 35487, USA; Department of Biological Sciences, The University of Alabama, Tuscaloosa, AL 35487, USA

**Keywords:** *Naegleria fowleri*, free-living amoebae, microbial ecology, abiotic, biotic, pathogen

## Abstract

*Naegleria fowleri* is a free-living protozoan that resides in soil and freshwater. Human intranasal amoebae exposure through water or potentially dust particles can culminate in primary amoebic meningoencephalitis, which generally causes death. While many questions remain regarding pathogenesis, the microbial ecology of *N. fowleri* is even less understood. This review outlines current knowledge of the environmental abiotic and biotic factors that affect the distribution and abundance of *N. fowleri*. Although the impacts of some abiotic factors remain poorly investigated or inconclusive, *N. fowleri* appears to have a wide pH range, low salinity tolerance and thermophilic preference. From what is known about biotic factors, the amoebae preferentially feed upon bacteria and are preyed upon by other free-living amoebae. Additional laboratory and environmental studies are needed to fill in knowledge gaps, which are crucial for surveillance and management of *N. fowleri* in freshwaters. As surface water temperatures increase with climate change, it is likely that this amoeba will pose a greater threat to human health, suggesting that identifying its abiotic and biotic preferences is critical to mitigating this risk.

## Teaser

*Naegleria fowleri*, the so-called brain-eating amoeba, is thermophilic, suggesting that its occurrence will increase with climate change, highlighting the need to better understand which abiotic and biotic factors influence its distribution and abundance.

## INTRODUCTION

*Naegleria fowleri*, referred to as the ‘brain-eating amoeba’, is the etiologic agent of primary amoebic meningoencephalitis (PAM). These microscopic, thermophilic, free-living amoebae are found in freshwater lakes, ponds and rivers (Stevens *et al*. 1977; Griffin 1983; Kyle and Noblet 1985; Detterline and Wilhelm 1991) as well as in soils and sediments (Wellings *et al*. 1977; Moussa *et al*. 2015). Pathogen exposure typically occurs through recreational water activities such as swimming or diving when water is forced up the nasal cavity (Heggie 2010). *Naegleria fowleri* infects human hosts by penetrating nasal mucosa and traveling from the nose to the brain via the olfactory nerve (Grace, Asbill and Virga 2015). When it reaches the brain, it produces inflammation culminating in PAM. Although contracting PAM is rare, the disease progresses rapidly and has a mortality rate between 95% and 99% (Heggie and Küpper 2017). Since *N. fowleri* was identified as the etiologic agent of PAM in 1966, there have been almost 400 confirmed PAM cases worldwide with only 7 survivors (Gharpure *et al*. 2020). However, PAM is thought to be dramatically underreported, with a recent estimate suggesting it was responsible for 16 deaths per year in the United States between 1999 and 2010 (Matanock *et al*. 2018), and cases are likely to increase as a result of climate change (Maciver, Piñero and Lorenzo-Morales 2020).

*Naegleria fowleri* exhibits three life stages, including a motile flagellate, feeding trophozoite and non-motile cyst. The flagellated form allows the organism to use its two flagella for locomotion while searching for food. When food is located, the flagellate transforms into the feeding trophozoite, which has sucker-like feeding cups called amoebastomes (John, Cole and Marciano-Cabral 1984). The trophozoite is typically considered the infectious form, but a previous study produced PAM upon nasal inoculation of flagellates, although the amoebae may have rapidly transitioned to the trophozoite form (Singh and Das 1972). Baig (2016) hypothesized that *N. fowleri* trophozoites are attracted by chemical neurotransmitters associated with neurons in the brain. Encystment occurs when *N. fowleri* faces adverse conditions, such as prolonged exposure (>72 h) to high temperatures (44°C), low-pH environments, intolerable salinity levels or ingestion of particular bacteria (Kadlec 1975; Fritzinger and Marciano-Cabral 2004; Lam, He and Marciano-Cabral 2019). Thus, environment impacts the distribution, abundance and life stage of *N. fowleri*, which has cascading implications for public health.

*Naegleria fowleri* persists in warm, moist environments, including both naturally heated and thermally polluted bodies of freshwater and soils (De Jonckheere and Van De Voorde 1977; Brown *et al*. 1983; Sykora, Keleti and Martinez 1983; Moussa *et al*. 2013, 2015). The amoeba has been detected in drinking water distribution systems, swimming pools, rainwater tanks, and tap and well water (Kadlec, Červa and Škvárová 1978; Blair *et al*. 2008; Puzon *et al*. 2009; Morgan *et al*. 2016; Waso *et al*. 2018). It is thought that *N. fowleri* may reside in pore spaces between soil particles and be introduced to surface waters during periods of rain via runoff (Singh 1975). Another potential dispersion mechanism is through dust particles transporting the cyst form by wind (Lawande *et al*. 1979; Lawande 1983; Ithoi *et al*. 2011), although Dorsch, Cameron and Robinson (1983) question this mechanism due to cyst sensitivity to desiccation. Strangely, *N. fowleri* has been cultured from the nasal passages of healthy individuals who did not develop PAM (Abraham and Lawande 1982; Rivera *et al*. 1984), raising questions about the intranasal microbial ecology and pathogenicity of the amoeba and/or immune response of the host.

Although the United States does not routinely test recreational waters for *N. fowleri*, the amoeba is listed as a contaminant under the US Environmental Protection Agency's (USEPA) Candidate Contaminant List, where it is not currently subject to EPA drinking water regulations (Bartrand, Causey and Clancy 2014; USEPA 2016). However, Australia, which reported the first *N. fowleri* death in 1965, imposed an action threshold of 2 *N. fowleri*/L of water (or detection in a 500-mL sample) and continued monitoring if the amoeba was detected (National Health and Medical Research Council 2018). Using a most probable number (MPN) approach, Lugg and Psaila-Savona (2001) had previously proposed a guideline limit of 5 thermophilic *Naegleria*/L of water and an actionable limit of 2 *Naegleria*/L of water for Western Australia. However, the MPN process is time consuming as it requires cultivation and has detection limitations. Thus, culture-independent methods, which rely on molecular identification and are more sensitive but do not distinguish between viable or dead amoebae, are becoming more prevalent (Mull, Narayanan and Hill 2013; Xue *et al*. 2018). Some environmental studies and monitoring programs employ a combination approach utilizing cultivation of *Naegleria* spp. followed by molecular analysis (Blair *et al*. 2008; Jamerson *et al*. 2009; Sifuentes *et al*. 2014; Morgan *et al*. 2016). Typically, polymerase chain reaction (PCR) or quantitative PCR (qPCR) is used to amplify genetic sequences unique to *N. fowleri*, such as portions of the 5.8S rRNA gene and ITS1 or ITS2 regions (Streby *et al*. 2015), a 166-bp fragment of the single-copy gene *Mp2Cl5*, which corresponds to an outer membrane protein present at each life stage (Reveiller, Cabanes and Marciano-Cabral 2002; Behets *et al*. 2007), or a 153-bp fragment of the 18S rRNA gene (Qvarnstrom *et al*. 2006). In a comparison of qPCR assays, Streby *et al*. (2015) found the 18S rRNA gene target provided the greatest accuracy and specificity with limited organism cross-reactivity from environmental samples (surface water and sediment) seeded with two of the eight described *N. fowleri* genotypes (De Jonckheere 2011), but the remaining genotypes have not been tested, indicating that more studies are needed to confirm this result. Although present as a single copy per amoeba, *Mp2Cl5* also displayed high specificity with a low detection limit (Behets *et al*. 2007; Streby *et al*. 2015). Quantification provides information on how ecological factors affect the number of *N. fowleri* in the environment, which could translate into an increased or decreased risk of human exposure. However, for having a worldwide distribution, there have been relatively few *N. fowleri* environmental surveys with attempts at quantification. Instead, presence/absence surveys prevail, likely due to limitations associated with cultivation, DNA extraction or cost.

Human cases of PAM have most frequently been reported from Australia, Pakistan, Czech Republic, India and the United States (Bartrand, Causey and Clancy 2014; Jahangeer *et al*. 2020). PAM cases have also been observed in mammals, especially cattle, in Brazil, Algeria and the United States (Visvesvara *et al*. 2005; Pimentel *et al*. 2012; Benterki *et al*. 2016). With the exception of Antarctica, *N. fowleri* has been detected on all continents (De Jonckheere 2011). Based on its thermophilic preference, most of the US cases have unsurprisingly been reported from southern-tier states. However, a PAM case in Minnesota may suggest range expansion (Kemble *et al*. 2012). *Naegleria fowleri* has also been detected in states that have not reported PAM cases, including Illinois, Alabama and Connecticut (Huizinga and McLaughlin 1990; Detterline and Wilhelm 1991; Maclean *et al*. 2004). Only a single study examined the spatial distribution of *N. fowleri* in US recreational waters, reporting the presence of thermotolerant amoebae at 34 of the 59 sites (57.6%) spanning 24 states, but *N. fowleri* was detected at only 6 sites (10.2%; Detterline and Wilhelm 1991). At these locations, estimated *N. fowleri* density was one amoeba per 500 mL with water temperatures ranging from 15.6°C to 23.9°C. In artificially heated water bodies in Florida and Texas, *N. fowleri* was detected at 3 of the 13 sites (23%) sampled, even though the amoeba was expected to thrive under these conditions (Stevens *et al*. 1977). Thus, despite the expansive distribution of *N. fowleri*, its presence seems sporadic and does not consistently overlap with PAM occurrences, hinting at a complex interaction of ecological abiotic and biotic factors.

## ABIOTIC FACTORS

Abiotic factors affecting the distribution and abundance of *N. fowleri* likely include temperature, light, pH, dissolved oxygen, salinity, chemical agents and nutrient availability. Current knowledge about the effect of each will be discussed (Table 1).

**Table 1.** Correlations between selected abiotic factors and *N. fowleri* from surveys of either natural environments (N) or engineered environments (E), where engineered environments include artificial thermal additions or discharges. DO is dissolved oxygen. Detection methods used in the studies include cultivation (C), morphological examinations (M), molecular (MO) assays with the target gene indicated, mouse pathogenicity test (P) and immunological techniques (I). For molecular approaches, RFLP is restriction fragment length polymorphism and ITS is internal transcribed spacer region. A ‘Yes’ indicates a correlation between the factor and *N. fowleri* and a ‘No’ indicates no correlation, with a '+' or '−' indicating the positive or negative nature of that correlation, respectively. Unspecified correlations are designated 'U' when the factor was measured but no conclusion drawn, and NA indicates that no measurements were taken. PN refers to pathogenic *Naegleria*, which at the time of this publication likely referred to *N. fowleri* as it was the only pathogenic species within the genus.

Citation	Sample type	Detection	Temp.	pH	Conductivity	DO	Turbidity	Chemical parameters
Blair *et al*. (2008)	Well water; E	C; MO (Mp2Cl5)	U	U	U	NA	U	U
Brown *et al*. (1983)	Thermal pool/springs; N and E	C; M; P; I	U	U	NA	NA	NA	U
Delattre and Oger (1981)	Water; sediment; N and E	C; P	U	U	NA	NA	NA	NA
Detterline and Wilhelm (1991)	Surface freshwater; N	C; M; enzyme analysis	No	NA	NA	NA	NA	No (dissolved iron)
Dobrowsky *et al*. (2016)	Water from tanks; E	MO (18S rRNA)	Yes (−)	No	NA	NA	NA	NA
Duma (1981)	Water and soil; N	C; P	Yes (+)	No	No	No	NA	NA
Fliermans *et al*. (1979)	Water; sediment; algal samples; N and E	C; M; P; I	U	U	NA	U	NA	NA
Huizinga and McLaughlin (1990)	Water; sediment; N and E	C; M; P; MO (DNA RFLP)	Yes (+)	NA	NA	NA	NA	NA
Issac and Sherchan (2020)	Rural drinking water distribution system (DWDS) water; E	MO (18S rRNA)	No	Yes (+)	Yes (+)	No	NA	Yes (salinity +)
Jamerson *et al*. (2009)	Surface water; sediment; N and E	C; MO (Mp2Cl5)	No	No	No	No	NA	NA
John and Howard (1995)	Surface water; swabs; N	C; M; P	U	U	NA	NA	NA	NA
Kemble *et al*. (2012)	Surface water; sediment; N	C; MO (18S rRNA)	NA	NA	NA	NA	NA	NA
Kim *et al*. (2018)	Water; N	C; MO (18S rRNA)	U	U	NA	NA	U	NA
Kyle and Noblet (1985)	Lake water (0–8.5 m); N	C; M; P	U	U	U	U	NA	U (iron trend)
Kyle and Noblet (1987)	Water (varied depth); N	C; M; P	U	U	NA	U	NA	NA
Lares-Villa and Hernandez-Pena (2010)	Freshwater; N	C; M; MO (ITS rRNA)	U	NA	NA	NA	NA	NA
Laseke *et al*. (2010)	Ground water aquifers (wells); E	MO (Mp2Cl5)	No	No	No	No	NA	NA
Maclean *et al*. (2004)	Pond water; rock/soil swabs; N	C; MO (Mp2Cl5)	U	NA	NA	NA	NA	NA
Morgan *et al*. (2016)	DWDS water; biofilm; E	C; MO (ITS rRNA)	No	NA	NA	NA	No	NA
Moussa *et al*. (2013)	Water; sediment; N and E	C; M; MO (ITS rRNA)	No	No	NA	NA	Yes (+)	No
Moussa *et al*. (2015)	Water; soil; N and E	C; M; MO (ITS rRNA)	NA	NA	NA	NA	NA	NA
Mull, Narayanan and Hill (2013)	Lake water; sediment; N	C and MO	NA	NA	NA	NA	NA	NA
Painter (2011); Painter *et al*. (2013)	Water (0 m; 0.3 m); N	MO (18S rRNA)	No	No	No	No	NA	Yes
Panda *et al*. (2015)	Lake/pond water; N	C; MO (ITS rRNA)	U	U	NA	NA	NA	NA
Puzon *et al*. (2009)	Drinking water; biofilm; E	C; MO (ITS rRNA)	NA	NA	NA	NA	NA	NA
Puzon *et al*. (2017)	DWDS biofilm; E	MO (ITS rRNA)	NA	NA	NA	NA	NA	NA
Sheehan *et al*. (2003)	Sediment; biofilms; water; N	MO (ITS rRNA)	U	U	NA	NA	NA	NA
Sifuentes *et al*. (2014)	Recreational surface water; N	C; MO (Mp2Cl5)	No	No	No	NA	No	NA
Stevens *et al*. (1977)	Surface water; N and E	C; M; P; I	U	NA	NA	NA	NA	NA
Sykora, Keleti and Martinez (1983)	Water; sediment; N and E	C; P	Yes (PN +)	No	No	NA	NA	NA
Tung *et al*. (2013)	Water; N and E	MO (18S rRNA)	No	No	NA	NA	No	NA
Tyndall *et al*. (1989)	Water; sediment; N and E	C; P	Yes (+)	U	U	U	NA	NA
Waso *et al*. (2018)	Roof-harvested rainwater tanks; E	MO (18S rRNA)	NA	NA	NA	NA	NA	NA
Wellings *et al*. (1977)	Water; sediment 5 ft from shore; N and E	C; M; P; I	U	NA	NA	NA	NA	NA
Xue *et al*. (2018)	Brackish surface water; N	MO (18S rRNA)	Yes (+)	No	No	Yes (−)	No	Yes (− trend for salinity)

### Temperature

*Naegleria fowleri* is considered to be a thermophilic amoeba (Griffin 1972; Cerva 1977), although strains differ slightly in their thermotolerance, and the organism may be more thermotolerant than thermophilic. The upper thermal limit for growth on slants was 46°C (Griffin 1972), but growth curve analyses showed temperature optima varied with culture media composition and concentration and inoculum vitality and size (Cerva 1977). At 20°C, amoebae replication did not occur, and shorter generation times (Cerva 1977) and greater activity (Griffin 1972) were noted as temperature increased to 40°C. *Naegleria fowleri* can persist for short durations (24–96 h) at water temperatures above 45°C (Lam, He and Marciano-Cabral 2019), with trophozoites and the more thermally resistant cysts surviving minutes to hours at 50°C–65°C (Chang 1978). Many researchers utilize their ability to survive at higher temperatures to preferentially isolate pathogenic *Naegleria*.

*Naegleria fowleri* thrives in warm waters, whether naturally or artificially heated (De Jonckheere, Van Dijck and Van De Voorde 1975; Sykora, Keleti and Martinez 1983; Tyndall *et al*. 1989). The amoeba has been detected in heated nuclear reactor effluent worldwide (De Jonckheere, Van Dijck and Van De Voorde 1975) but can also persist in relatively cold water (Wellings *et al*. 1977). Cysts survived temperatures below 10°C (Chang 1978; John 1982), and frozen cysts were able to excyst after 8 months of storage and maintain virulence (Warhurst, Carman and Mann 1980). Cysts have also been shown to overwinter in lake sediments and re-emerge when conditions become favorable, which suggests a sustained presence at sites with previous *N. fowleri* detection (Wellings *et al*. 1977).

From 1962 to 2018, there were increases in US PAM cases from June to September compared with other months (CDC 2020). This trend may be due to increased recreational water activity and subsequent *N. fowleri* exposure rather than increased amoebae presence as there is no consensus on the impact of seasonality on *N. fowleri* presence or abundance. Several studies reported no significant seasonal correlation (Lares-Villa and Hernández-Peña 2010; Sifuentes *et al*. 2014), while another found a significant correlation, with higher concentrations of amoebae during summer than winter (Xue *et al*. 2018). Although not significant, Sifuentes *et al*. (2014) detected *N. fowleri* more frequently in winter and spring (20%) samples than in summer and fall (7.9%) samples, suggesting that there are likely multiple factors that influence the occurrence of this amoeba. In a *N. fowleri* colonized drinking water distribution pipeline, amoebae concentrations in bulk water were highest when water temperatures peaked, and the amoebae transitioned to pipe wall biofilms when water temperatures decreased (Puzon *et al*. 2020). Other studies were only able to detect *N. fowleri* from environmental water samples during warmer months (John and Howard 1995; Lares-Villa and Hernández-Peña 2010; Panda *et al*. 2015), suggesting that water temperature may be more influential than seasonality, especially as temperature influences whether the amoeba may be free-living or biofilm-associated.

In cultivation-based field studies, Huizinga and McLaughlin (1990) found a correlation between temperature and *N. fowleri* presence, while several other studies reported no correlation but were either conducted at the same lake for a short duration (e.g. three sampling periods over 4 months; Jamerson *et al*. 2009) or at multiple locations for a single sampling period (Detterline and Wilhelm 1991) and reported a low overall prevalence of *N. fowleri*. Molecular genetic methods may allow for a more robust quantification of the organism at various temperatures than cultivation-based approaches, although a combined approach of cultivation followed by molecular analyses provides more information on organism presence as well as viability (Kim *et al*. 2018). However, using molecular methods, Xue *et al*. (2018) demonstrated a correlation between *N. fowleri* presence and temperature.

*Naegleria* *fowleri* has been detected in environmental water samples from 16°C to 47°C (Sykora, Keleti and Martinez 1983; Blair *et al*. 2008; Jamerson *et al*. 2009; Laseke *et al*. 2010; Kim *et al*. 2018; Xue *et al*. 2018) and was recovered from sediments at 12°C (Wellings *et al*. 1977), highlighting the importance of surveillance over a wide range of water temperatures rather than focusing solely on elevated temperatures. While temperature plays a role in *N. fowleri* persistence, it cannot account for *N. fowleri* distribution patterns alone, as evidenced by its sporadic presence (Delattre and Oger 1981). Detterline and Wilhelm (1991) contend temperature acts as a mechanism to clear potential ecological competitors, allowing *N. fowleri* proliferation, but this is still to be determined. Teasing apart temperature complexities relating to the distribution and abundance of *N. fowleri* will require both increased environmental surveillance and laboratory studies. Cerva (1977) found that growth temperature optima changed with media composition, suggesting nutrient availability paired with temperature affects *N. fowleri* growth. Thus, the synergistic effects of temperature and other abiotic or biotic factors need to be investigated.

### pH

Environmental studies have reported no correlation between pH and *N. fowleri* presence and abundance (Detterline and Wilhelm 1991; Jamerson *et al*. 2009; Laseke *et al*. 2010; Xue *et al*. 2018). Under laboratory conditions, the amoeba persists from pH 2.0 to 8.15 (Sykora, Keleti and Martinez 1983), with an optimal pH of 6.5 (Cerva 1978). In freshwater with pH below 2, *N. fowleri* becomes immediately non-viable and becomes non-viable within 72 h at pH 3 but persists at least 96 h at pH values of 4–11 (Lam, He and Marciano-Cabral 2019). At a pH of 12, *N. fowleri* are viable for ∼24 h and rapidly lose viability with increasing pH (Lam, He and Marciano-Cabral 2019). John and Howard (1995) isolated *N. fowleri* from water samples with a range of pH from 5.1 to 8.0, Panda *et al*. (2015) from water with pH 6.3 and Kim *et al*. (2018) from water with pH 7.8 and 8.2. Thus, pH values typically found in recreational waters do not seem to be a limiting factor for *N. fowleri* distribution (Delattre and Oger 1981; Kyle and Noblet 1985). However, the potential interactive effect of pH with other abiotic and biotic factors remains to be explored.

### Light

The effect of light on growth and survival of *N. fowleri* has not been well studied, but the amoebae are fairly resistant to inactivation by ultraviolet irradiation (Sarkar and Gerba 2012). Light may indirectly influence *N. fowleri* by increasing temperature, which may promote metabolic activity or increase the availability of prey. All else being equal, water bodies receiving direct sunlight likely provide a more suitable habitat than shaded water bodies, but this has not been confirmed. Because *N. fowleri* persists in water pipelines and soils and sediments that receive little to no sunlight (Moussa *et al*. 2015; Puzon *et al*. 2017), it is unlikely that sunlight plays a role in determining amoebae presence, but no studies have investigated whether sunlight impacts *N. fowleri* proliferation, life stage regulation or microbial prey communities.

### Salinity

*Naegleria fowleri* thrives in freshwater, persists in brackish water and has not been detected in marine environments (Xue *et al*. 2018), likely because pathogenic *Naegleria* becomes immobilized by 3% NaCl or sterilized sea water (Carter 1970). The salinity tolerance of this amoeba varies somewhat with strains, with some tolerating 0.5%–1.0% NaCl salinity and a few being inhibited by a low percentage of a combination of salts (e.g. 0.2% NaCl and KCl) (Kadlec 1975; Griffin 1983). The *N. fowleri* inhibition previously reported on agar containing 0.5% NaCl also occurred in liquid media, with a similar inhibitory effect associated with KCl, and dissolved inorganic salts affected amoebae generation times (Cerva 1978). Salinity and CaCl_2_ also induce encystment (Kadlec 1975), with a recent study demonstrating that *N. fowleri* trophozoites encyst or become non-viable more rapidly with increasing salinity (maximum salinity tested was 1.6% and maximum time of viability tested was 96 h; Lam, He and Marciano-Cabral 2019). *Naegleria fowleri* was detected in brackish surface water (0.01%–0.24%), with a negative but non-significant correlation between *N. fowleri* abundance and salinity (Xue *et al*. 2018). Thus, the distribution of *N. fowleri* appears to be regulated by salinity, with the amoebae able to tolerate salinity to ∼1.6% for a short duration. However, laboratory settings do not include other environmental factors or stressors, highlighting the need for factorial experiments and more surveys of brackish waters. Regardless, the viability of *N. fowleri* for short durations at higher salinity suggests that brackish waters in coastal environments may be a source for human exposure.

### Conductivity

Statistically significant correlations of conductivity with *N. fowleri* presence or abundance were not found by Laseke *et al*. (2010) or Jamerson *et al*. (2009) but were detected by Painter (2011). Because conductivity encompasses different ion compositions, developing a realistic conductivity assay for environmental samples may be challenging and would likely require varied ratios of ion compositions representing a range of environmental conditions. Different ions and ion concentrations may affect *N. fowleri* differently depending on its metabolic pathways, but more investigation is needed.

### Turbidity

Few environmental surveys for *N. fowleri* have measured turbidity. Of those that did, although the amoebae were detected across a wide range of NTU values, the majority did not find a correlation between turbidity and *N. fowleri* presence or abundance in recreational surface fresh or brackish waters, drinking water or well water (Blair *et al*. 2008; Sifuentes *et al*. 2014; Morgan *et al*. 2016; Xue *et al*. 2018). However, Moussa *et al*. (2013) reported a significant positive correlation between *N. fowleri* numbers and turbidity from a survey of geothermal water and sediment. Turbidity can be influenced by suspended sediments or elevated nutrient inputs, which may increase potential prey availability for free-living amoebae that can attach to particulate matter to graze on biofilms (Preston and King 2003). Turbidity is also affected by precipitation that resuspends particulates in the water column or introduces particulate material, including *N. fowleri*, from the surrounding environment into water bodies (Brown *et al*. 1983; Moussa *et al*. 2013, 2015). Although Painter *et al*. (2013) did not detect *N. fowleri* in samples following a precipitation event, Kyle and Noblet (1987) did find a significant increase in *Naegleria* spp. after precipitation. Additional studies of turbidity and *N. fowleri* presence before and after precipitation events are needed to evaluate the effect of precipitation and resulting runoff on *N. fowleri* abundance. If surveillance reveals higher *N. fowleri* abundance or prevalence after precipitation events, this information could be used to better predict favorable environmental conditions for the amoeba.

### Spatial distribution and depth

Increasing water column depth may influence *N. fowleri* presence due to altered microclimate variables such as decreased water temperature, changes in dissolved oxygen or decreased ultraviolet light exposure, but the effect of depth on the distribution of the amoeba is understudied. Painter *et al*. (2013) found no significant difference in *N. fowleri* abundance between surface (0 m) and subsurface (0.3 m) water samples, but a study sampling various depths within two monomictic lakes reported a significant positive correlation of depth with thermotolerant amoebae, where *N. fowleri* presence may have been due to the availability of exogenous iron (Kyle and Noblet 1985).

While *N. fowleri* vertical distribution within the water column is relatively unexplored, several studies examined amoebae presence within bulk water and sediment samples. Although different sample volumes were used for water and sediment analyses, these studies found no significant difference in *N. fowleri* abundance or presence between water and sediment samples (Delattre and Oger 1981; Huizinga and McLaughlin 1990; Jamerson *et al*. 2009; Moussa *et al*. 2013). Future studies using a standardized sampling volume would be beneficial for the evaluation of *N. fowleri* abundance in water and sediment although this may also introduce inhibitors that impact detection abilities, particularly for molecular genetic approaches.

Distribution of *N. fowleri* in water with increasing distance from shore has not been evaluated. This is likely due to the relatively low presence or abundance of *N. fowleri* within most sampled water bodies. Because the amoeba can transform into a motile flagellate, the presence of *N. fowleri* may be more relevant than its spatial distribution for risk assessment, especially with the low risk of contracting *N. fowleri* from recreational surface water (Cabanes *et al*. 2001). However, understanding the horizontal distribution of *N. fowleri* is important because people swim near shores due to accessibility. Shores also accumulate debris from wind movement across water bodies, which may provide biofilms for *N. fowleri* to prey on, and are where runoff, potentially including the amoeba, is introduced into the water body.

### Dissolved oxygen

*Naegleria fowleri* is an aerobic organism that uses dissolved oxygen in water (Martinez *et al*. 1973; Schuster and Rechthand 1975; John 1982). Laboratory respiration studies found that under agitated axenic culture conditions, *N. fowleri* consumed 30 ng O/min/mg of cell protein during log growth (Weik and John 1979; John 1982). Compared with the non-pathogenic *Naegleria gruberi*, *N. fowleri* consumed substantially less oxygen, which led researchers to hypothesize that this lower oxygen demand enhanced the ability of *N. fowleri* to thrive in heated waters where dissolved oxygen concentrations are reduced (John 1982).

Although *N. fowleri* needs oxygen for energy generation, it has been isolated from anaerobic sediments (Wellings *et al*. 1977), suggesting that anoxic conditions do not kill all life stages of the amoeba. Of the studies that measured dissolved oxygen in the ambient water, no uniform trend regarding correlation with *N. fowleri* presence could be determined. In several studies, dissolved oxygen concentrations did not correlate with *N. fowleri* presence (Jamerson *et al*. 2009; Laseke *et al*. 2010; Painter 2011; Painter *et al*. 2013). However, Xue *et al*. (2018) reported a significant negative correlation between *N. fowleri* abundance and dissolved oxygen although this may have been due to the significant negative correlation between water temperature and dissolved oxygen. Kyle and Noblet (1985) also found a significant negative correlation between free-living amoebae abundance and dissolved oxygen but did not examine *N. fowleri* presence specifically. The concentration of dissolved oxygen can be influenced by various factors, suggesting that these negative correlations require further study, especially in factorial experiments paired with different water temperatures.

### Nutrient availability

Nutrient availability impacts organism growth, replication and biomass. A minimal medium for cultivation of *N. fowleri* includes amino acids (arginine, glycine, histidine, isoleucine, leucine, methionine, phenylalanine, proline, threonine, tryptophan and valine), vitamins (biotin, folic acid, hemin, pyridoxal, riboflavin and thiamine), guanosine, glucose, salts and metals (Nerad, Visvesvara and Daggett 1983). Specifically, iron has been proposed as a necessary nutrient for *N. fowleri* (Newsome and Wilhelm 1981; Kyle and Noblet 1985), and Duma (1981) suggested that manganese may also be beneficial due to amoebae isolation from areas with relatively high manganese concentrations. While iron may be essential, a correlation between the availability of dissolved iron and *N. fowleri* presence in environmental waters was not found (Detterline and Wilhelm 1991). Laboratory studies found some iron-containing porphyrins enhanced *N. fowleri* viability and pathogenicity and influenced amoeba growth and mobility although a non-iron-containing porphyrin also increased pathogenicity and mobility (Bradley *et al*. 1996). Similarly, *N. fowleri* growth was inhibited by iron-chelating compounds derived from microbes, and the addition of iron reduced this inhibition (Newsome and Wilhelm 1983). Iron chelation was reduced at higher temperatures, leading Newsome and Wilhelm to hypothesize that the reduction in microbial iron chelation at elevated water temperatures promoted *N. fowleri* persistence. Although an association with iron has been suggested, the importance of iron for the microbial ecology of *N. fowleri* in natural systems needs further investigation.

Few environmental surveys for *N. fowleri* detection included nutrient analyses (Painter 2011, 2013; Moussa *et al*. 2013). Moussa *et al*. (2013) surveyed geothermal recreational water in Guadeloupe for K, Ca, Mg, Na, SiO_2_, total organic carbon, permanganate oxidation, NH_4_, NO_3_ and HCO_3_ and found that while chemical concentrations varied within and among sites, there was no correlation of these chemicals with *N. fowleri* presence. Painter (2011) measured NO_3_, OPO_4_, Cl and SO_4_ in lake surface water samples and found a significant negative correlation of *N. fowleri* abundance with concentrations of NO_3_ and OPO_4_, and a significant positive correlation with concentration of SO_4_ and the number of days since last precipitation (Painter 2011). In subsurface samples, there was also a significant negative correlation of *N. fowleri* abundance with NO_3_ and a positive correlation with days since last precipitation. However, these results should be interpreted cautiously as no *N. fowleri* were detected in stormflow samples, yet stormflow values largely drove the significant correlations for both surface and subsurface samples (Painter 2011). More studies that incorporate nutrient analyses are needed, especially when precipitation events occur and free-living amoeba (FLA) in soils may be introduced to water bodies. By studying nutrient concentrations in environmental water bodies and determining the presence of *N. fowleri*, patterns may be discerned, which could aid future management strategies and indicate whether anthropogenic activities such as nutrient enrichment or pollution influence pathogen presence.

### Chemical agents

The presence of *N. fowleri* can be affected by the addition of chemical agents such as chlorine, which is added to swimming pools and drinking water pipelines to discourage microbial growth. Even with low but measurable chlorine residual or chloramine addition, *N. fowleri* has been detected in swimming pools and drinking water distribution pipelines (Cursons *et al*. 1979; Miller *et al*. 2015, 2017; Morgan *et al*. 2016; Puzon *et al*. 2017). Because chlorine levels in drinking water decreased with distance from the chlorine treatment point, distance from the chlorine treatment point was positively correlated with *N. fowleri* presence (Morgan *et al*. 2016). Biofilm formation also contributed to *N. fowleri* survival in the presence of chlorine residual because biofilm matrices and polymers prevented chlorine dissolution, acting as a physical buffer from the chemical agent (Miller *et al*. 2015; Morgan *et al*. 2016). However, Miller *et al*. (2017) demonstrated *N. fowleri* elimination from drinking water distribution system (DWDS) water and biofilm with adequate chlorination.

### Environmental disturbance

While limited studies explore this possibility, environmental disturbance has been proposed to promote *N. fowleri* growth (Griffin 1983). This coincides with the flagellate-empty hypothesis, which suggests man-made or natural disturbances remove competitors and the ability of *N. fowleri* to transform into a flagellate confers an advantage in recolonizing disturbed environments (Griffin 1983). John (1982) noted *N. fowleri* appear to particularly thrive in man-made environments and Detterline and Wilhelm (1991) reported a significant positive correlation between environmental disturbance and *N. fowleri* presence in a survey of US recreational waters. Additional research in this area is needed and could focus on determining the mechanism(s) by which disturbance impacts *N. fowleri*.

### Water availability

*Naegleria fowleri* requires moisture for survival, which is evident by the organism's sensitivity to desiccation (Chang 1978). Drying renders trophozoites immediately non-viable and cysts non-viable in <5 min (Chang 1978). However, a PAM case has been attributed to inhalation of dust-borne cysts, which suggests cysts can survive with limited moisture for longer durations (Lawande *et al*. 1979). More research into cyst survival is needed.

## BIOTIC FACTORS

Biotic factors impacting the distribution and abundance of *N. fowleri* include presence of predators, prey availability and identity, competition, inclusion in biofilms, interactions and correlations with other organisms, and chemical signaling. Current knowledge about the influence of each is discussed (Table 2).

**Table 2.** Association of *N. fowleri* with other organisms.

Citation	Organism(s)	Relationship to *N. fowleri*
Dobrowsky *et al*. (2016)	*Legionella* spp.; *Acanthamoeba* spp.; *Vermamoeba*(*Hartmanella*)*vermiformis*	Co-occurring (significant + correlation)
Fritzinger and Marciano-Cabral (2004)	*Pseudomonas aeruginosa*	Increased CD59-like protein expression upon exposure
Galvez *et al*. (1993); Lebbadi *et al*. (1995)	*Bacillus licheniformis* strains	Chemical signaling, inhibition
Griffin (1983)	*Naegleria lovaniensis*	Competitor
Issac and Sherchan (2020)	Enterococci	Co-occurring (+ correlation)
Marciano-Cabral and Cline (1987)	*Escherichia coli*;*Bacillus megaterium*;*Pseudomonas aeruginosa*	Prey
Miller *et al*. (2018a)	*Meiothermus ruber*;*Meiothermus chliarophilus*	Prey
Morgan *et al*. (2016)	Bacterial richness	Significant + correlation
Morgan *et al*. (2016); Puzon *et al*. (2017)	Cytophagia; Planctomycetia; Alphaproteobacteria; Deltaproteobacteria; Deinococci (*Meiothermus*); Saprospirae; Nitrospira	Co-occurring (significant + correlation)
Morgan *et al*. (2016)	*Stenostomum*; *Monogononta*; *Meiothermus* spp.	Co-occurring (significant + correlation)
Newsome and Wilhelm (1983)	*Streptomyces pilosus*;*Rhodotorula pilimanae*	Derived compounds inhibit *N. fowleri*
Newsome *et al*. (1985)	*Legionella pneumophila*	Putative intracellular replication in *N. fowleri*
Paul, Ahmad and Sharma (2010)	*Proteus* spp.	Prey
Plasson and Mameri (2018)	*Willaertia magna*	Predator
Puzon *et al*. (2017)	*Philodinidae*;*Nematoda*	Co-occurring (+ correlation)
Rizo-Liendo *et al*. (2020)	*Streptomyces sanyensis*	Derived indolocarbazoles inhibit *N. fowleri* and induce cell death
Schuster (2002)	*Escherichia coli*;*Klebsiella pneumoniae*;*Enterobacter* spp.	Prey
Stevens, DeJonckheere and Willaert (1980)	*Naegleria lovaniensis*	Co-occurring
Tapia, Torres and Visvesvara (2013)	*Balamuthia mandrillaris*	Predator
Xue *et al*. (2018)	*Escherichia coli*	Co-occurring (significant + correlation)

### Presence of predators

Relatively little is known about organisms that prey on *N. fowleri*. Other free-living amoebae, including *Balamuthia mandrillaris* and *Willaertia magna*, were shown to ingest *N. fowleri* (Tapia, Torres and Visvesvara 2013; Plasson and Mameri 2018), and rotifers and other microbes present in lake water appeared to prey on *N. fowleri* (Jamerson *et al*. 2009). Rotifers have been shown to ingest protozoal cysts (Fayer *et al*. 2000; Trout, Walsh and Fayer 2002), providing additional support for this theory. Scheid (2015) suggested that amoebophagous fungi may prey on FLA, but studies on *N. fowleri* and other pathogenic FLA are lacking. Avoidance of predators and/or competitors was the basis for the flagellate-empty hypothesis proposed by Griffin (1983) to explain the paucity of *N. fowleri* recovered from unperturbed environments. Additional research into predation on *N. fowleri* is needed to better understand the distribution patterns of this pathogenic amoeba and potential biocontrol mechanisms. Future studies could also investigate how predator presence affects *N. fowleri* life stage differentiation or pathogenicity.

### Availability and identity of prey

The vertical distribution of *N. fowleri* in freshwater lakes was shown to be impacted by prey availability (Kyle and Noblet 1985). As bacterivores, Kyle and Noblet (1985) found more *N. fowleri* in water layers containing filamentous bacteria, particulates and detritus. The amoebae can rapidly transform from a feeding trophozoite into a motile flagellate, allowing it to respond to prey availability. The non-pathogenic species *N. gruberi* transformed into the trophozoite form at the water–air interface to feed, suggesting that *N. fowleri* may also have this capability (Preston and King 2003) but this remains to be tested.

In laboratory cultivation studies, researchers typically use lawns of *Escherichia coli*, *Klebsiella pneumoniae* or *Enterobacter* spp. on non-nutrient agar as amoebae prey (Schuster 2002). During a laboratory prey experiment, a *Proteus* sp. was also partially consumed while *Pseudomonas pyocyanea* was not ingested by *N. fowleri* (Paul, Ahmad and Sharma 2010). Non-mucoid, Gram-negative bacterial strains seem to be preferred by *N. fowleri* (Schuster 2002), but differential ingestion of biofilm bacteria suggests that the amoebae display preferential grazing (Miller *et al*. 2018a). This preference may be due to pigment formation and exotoxin production by some bacterial species, both Gram-negative and Gram-positive, that protect them from ingestion and/or digestion by FLA (Singh 1942, 1975; Groscop and Brent 1964). Singh (1945) reported that soil amoebae prey upon bacteria discriminately and while amoebae showed no preference between Gram-negative and Gram-positive bacteria, there was less consumption of pigmented bacteria. This preference can be altered, however, as Chang (1960) reported *N. gruberi* tolerance to a yellow-pigmented *Flavobacterium* sp. after five subcultures. Nonetheless, the effect of pigment and ingestion/digestion of these bacteria by *N. fowleri* remains untested. Although *N. fowleri* may not be able to digest some bacteria, Marciano-Cabral and Cline (1987) showed that *N. fowleri* migration was stimulated by the presence of both Gram-negative and Gram-positive bacteria and was impacted by prey density. Danso and Alexander (1975) reported no *Naegleria* sp. growth when bacterial numbers fell to ∼10^7^ cells per mL although Goudot *et al*. (2012) found that biofilm-associated *N. fowleri* needed 10^4^ bacterial cells per amoeba for growth. Thus, more experiments are necessary to identify the chemical cues that *N. fowleri* responds to, their preferred prey items and required prey density. This understanding is important because associations with bacteria can influence FLA virulence and pathogenicity, as demonstrated in *Entamoeba histolytica* (Bhattacharya *et al*. 1998).

Due to their consumption of bacteria, several environmental surveys sought to examine correlations between *N. fowleri* and *E. coli* or other bacterial prey. Multiple studies employing molecular detection methods for the amoebae and cultivation approaches for the bacteria found no correlation between the abundance and/or presence of *N. fowleri* and *E. coli* (Painter *et al*. 2013; Sifuentes *et al*. 2014; Waso *et al*. 2018). However, a recent survey reported a significant positive correlation between *N. fowleri* and *E. coli* when using molecular approaches for both organisms but not when MPN methods were employed for *E. coli* quantification (Xue *et al*. 2018). This inconsistency suggests that additional studies into possible correlations with *E. coli* are warranted. Correlations between *N. fowleri* and *Enterococci*, a genus of Gram-positive bacteria, were not found (Waso *et al*. 2018; Xue *et al*. 2018) but the presence of coliform bacteria has been linked to increased *Naegleria* isolation (Brown *et al*. 1983; Sykora, Keleti and Martinez 1983). These coliforms likely serve as prey, but the influence of fecal coliforms on the growth of *N. fowleri* and potential for correlation requires further research.

Prey factors such as size, shape, composition, toxin production and chemical signaling affect consumption by FLA. A *Naegleria* sp. was shown to consume filamentous cyanobacteria but not aggregated cyanobacteria, but when sonicated fragments of the aggregates were available, they were ingested, suggesting that morphology plays a role in prey selection (Xinyao *et al*. 2006). Once prey has been ingested, the organisms can be excreted without being digested (Xinyao *et al*. 2006) or are lysed by naegleriapores or pore-forming proteins (Herbst *et al*. 2002). Some lysed bacteria secrete toxins and *N. fowleri* previously responded by increasing the expression of a CD59-like protein in the presence of these bacteria (Fritzinger and Marciano-Cabral 2004). Despite increased CD59-like protein expression, Fritzinger and Marciano-Cabral (2004) demonstrated that *N. fowleri* still ingested *P. aeruginosa*, which resulted in encystment and vacuole formation. Thus, additional studies are needed to assess *N. fowleri'*s global transcriptional response to seemingly harmful bacteria and determine whether isolate recovery or adaptation occurs.

### Competition

Competition from other FLA, including other thermophilic species, for resources may impact the distribution and abundance of *N. fowleri* in the environment. Within the genus *Naegleria*, *N. fowleri* can be found with *N. lovaniensis*, which is considered an ecological competitor due to co-occupancy of similar environmental niches (Stevens, DeJonckheere and Willaert 1980; Griffin 1983; Moussa *et al*. 2013). However, although Miller *et al*. (2018b) demonstrated viable *N. fowleri* and *N. lovaniensis* can temporarily co-occur under laboratory conditions, field samples from DWDS only detected viable *N. fowleri* and *Vermamoeba vermiformis* in a single sample and no co-occupancy of viable *N. fowleri* and *N. lovaniensis* was noted. *Naegleria fowleri* has a relatively slow growth rate compared with other amoebae, which reduces its ability to compete with other organisms in the environment (Griffin 1983). However, Puzon *et al*. (2009) found that biofilms containing various FLA changed composition over time, going from a mix of non-pathogenic species of FLA in March to only *N. fowleri* in May. In over 88% of water distribution system biofilms examined, only a single viable FLA was present using both cultivation and molecular detection methods (Miller *et al*. 2018b), suggesting that FLA do not effectively co-colonize biofilms, yet dominance of amoebae can change over time so dual colonization does occur (Puzon *et al*. 2009, 2017; Miller *et al*. 2018a,b). Thus, the flagellate-empty habitat hypothesis, which suggests *N. fowleri* tends to proliferate in environments where natural events or human intervention has removed usual competitors (Griffin 1983), warrants additional investigation, particularly in biofilms.

In addition to competing for prey, *N. fowleri* may also compete for less abundant minerals or vitamins within the environment. Kraft and Angert (2017) suggest that competition for thiamine structures ecological interactions and facilitates pathogenicity. Confirmed thiaminase 1 producers include *N. gruberi* (Kreinbring *et al*. 2014), and *N. fowleri* has a closely related sequence (Kraft and Angert 2017). A recent study on a thiaminase 1-producing *Burkholderia thailandensis* strain found a growth advantage was conferred to the bacteria by salvaging precursors from environmental thiamine and its analogs (Sannino *et al*. 2018). However, production of thiaminase 1 by *N. fowleri* has not been confirmed, warranting investigation into its potential for impacting microbial competition in the environment.

### Inclusion in biofilms

Despite chlorination efforts, *N. fowleri* has been isolated from biofilms in water distribution systems (Morgan *et al*. 2016; Miller *et al*. 2017, 2018b) and was detected from biofilms significantly more often than from bulk drinking water (Miller *et al*. 2018b). Besides providing protection from chemical disinfectant agents such as free residual chlorine, biofilms provide *N. fowleri* with a bacterial food source. Laboratory analysis of freshwater biofilms suggests that in addition to elevated temperature, *N. fowleri* requires 10^4^ bacteria per amoeba for growth, with a maximum growth rate reported at 10^6^ to 10^7^ bacteria per amoeba (Goudot *et al*. 2012). Increased bacterial richness, as well as a higher abundance of *Alphaproteobacteria*,*Saprospirae* and *Nematoda*, was also found to affect the growth of *N. fowleri* (Puzon *et al*. 2017). Availability of prey items may impact which FLA species can persist, as Morgan *et al*. (2016) reported a significant co-occurrence of members of the bacterial genus *Meiothermus* with *N. fowleri* and Miller *et al*. (2018a) found *N. fowleri* only in biofilms where *Meiothermus* spp. were found. This thermophilic, Gram-negative genus employs adhesion organelles for biofilm formation (Raulio *et al*. 2008), and *N. fowleri* has been shown to prey upon several species of *Meiothermus* (Miller *et al*. 2018a). However, more research is necessary to understand the microbial interactions that regulate biofilm formation and *N. fowleri*’s role within this microenvironment. Besides focusing on drinking water distribution pipelines, which likely have fewer environmental stressors and microbial additions, assessing the presence of *N. fowleri* in pond and/or lake biofilms will also be key to understanding its microbial interactions.

### Interactions or correlations with other organisms

Studies of *N. fowleri* presence with other organisms have allowed researchers to learn more about preferred associations and potential interactions. Many of these studies have focused on biofilms or bulk water within drinking water distribution pipeline systems, so less is known about associations or interactions that occur in natural systems such as lakes, ponds or geothermal springs. In warm groundwater aquifers, *N. fowleri* presence was correlated with diverse bacterial communities and *N. fowleri* was absent in samples rich in *Caldimonas* and *Leptothrix* spp. (Laseke *et al*. 2010). Similarly, in DWDS, *N. fowleri* absence was correlated with low bacterial richness but high bacterial richness was not itself predictive of *N. fowleri* presence (Morgan *et al*. 2016; Puzon *et al*. 2017), suggesting that more work is needed to investigate potential correlations with increased bacterial diversity. In DWDS, a number of bacterial clades were found to be significantly positively correlated with *N. fowleri* presence in bulk water and/or biofilms, including Cytophagia, Planctomycetia, Alphaproteobacteria, Deltaproteobacteria, Deinococci (*Meiothermus*), Saprospirae and Nitrospira (Morgan *et al*. 2016; Puzon *et al*. 2017). Correlations with the presence of Sphingomonadales were ambiguous, as Morgan *et al*. (2016) reported a negative correlation with *N. fowleri* presence, while Puzon *et al*. (2017) reported a positive correlation. As Delafont *et al*. (2013) found that Sphingomonadales were highly represented inside various FLA, its occurrence with *N. fowleri* warrants additional investigation. In harvested rainwater tanks, *N. fowleri* was significantly positively correlated with the presence of *Legionella* spp. (Dobrowsky *et al*. 2016) and both *L. pneumophila* and *N. fowleri* were found at the highest concentrations in lake biofilms during the summer (Żbikowska *et al*. 2014). While *N. fowleri* largely feeds on Gram-negative bacteria, which many of the co-occurring bacteria are, it is uncertain whether these significant co-occurrences represent predator–prey relationships or whether the organisms prefer the same microhabitat as members of many of the positively correlated bacterial clades are known to be thermotolerant or thermophilic.

Several eukaryotic taxa were also correlated with *N. fowleri* presence in biofilms. Morgan *et al*. (2016) reported significant co-occurrence of *N. fowleri* with *Stenostomum*, a family of freshwater flatworms, and *Monogononta*, a class of rotifers. The identification of a class of rotifers as positively correlated with *N. fowleri* presence is interesting, as rotifers have been hypothesized to prey on *Naegleria* (Jamerson *et al*. 2009). Puzon *et al*. (2017) also reported a rotifer class, *Philodinidae*, was more often found with *N. fowleri*, as were members of the phylum *Nematoda*, and reported that different FLA were found in biofilms with distinctive eukaryotic communities. As a result, Puzon *et al*. (2017) postulated that these phylogenetically distinct taxa may be useful indicators for the presence of specific FLA, including *N. fowleri*. Interestingly, more than one viable FLA was not observed in most biofilm and bulk water samples from DWDS (Morgan *et al*. 2016; Puzon *et al*. 2017; Miller *et al*. 2018b), suggesting that the presence of compatible organisms may dictate the presence of FLA. However, using molecular methods that do not discriminate between viable and non-viable organisms, Dobrowsky *et al*. (2016) reported significant positive correlations between the presence of *N. fowleri* and *Acanthamoeba* spp. and *Vermamoeba*(*Hartmanella*)*vermiformis* in harvested rainwater tanks. Thus, more studies are needed to tease apart these relationships, especially in natural environments.

*Naegleria fowleri* associations with fungi, viruses, algae and archaea are largely unexplored. Because soil is a reservoir for *N. fowleri* (Moussa *et al*. 2013, 2015), soil-dwelling fungi likely impact the amoeba by affecting bacterial prey availability through the production of antibacterial metabolites or nutrient availability from fungal decomposition processes. Puzon *et al*. (2017) found more fungi in DWDS biofilms that did not host *Naegleria* species, indicating that more research on potential interactions is warranted. Amoebae have also been shown to harbor viruses (La Scola *et al*. 2003). While no intracellular viruses have been isolated from *N. fowleri* or *Naegleria* species, virus-like particles within *N. gruberi* were found (Schuster 1969). Although *Naegleria* species and freshwater algae occupy similar environments, co-occurrence patterns and interactions are unknown. Similarly, no one has investigated interactions or associations of *N. fowleri* with archaea. Learning more about these associations and interactions may provide information regarding the pathogenicity, reproduction or resistance to environmental stressors demonstrated by *N. fowleri*.

### Chemical signaling

Microbial interactions include chemical signaling that can attract or repel *N. fowleri*. For example, *Bacillus licheniformis* strains were shown to exhibit antiamoebic effects on *N. fowleri* (Galvez *et al*. 1993; Lebbadi *et al*. 1995), and amoebicins effective against *N. fowleri* were identified in strain D-13 (Galvez *et al*. 1994). Because *Bacillus* spp. are typically found in soil, this ecological interaction supports the soil being a habitat for *N. fowleri*. Singh (1942) reported that several bacteria, including *Serratia marcescens*, produced toxic metabolic products that prevented soil-dwelling FLA from ingesting the bacteria. Marciano-Cabral and Cline (1987) investigated *N. fowleri* chemoattraction to *E. coli*, *P. aeruginosa* and *Bacillus megaterium*, and found that *N. fowleri* responded to bacteria via chemotaxis and food cup formation, and extended pseudopods toward ingestible bacteria. Marciano-Cabral and Cline (1987) also showed *N. fowleri* demonstrated a preference for bacterial extracts rather than nerve cell extracts. However, the extent of microbial interactions from chemical signaling with *N. fowleri* is largely unknown. While these interactions are ecologically relevant, studies may also reveal potential compounds or biological control mechanisms that could be used to regulate the presence and pathogenic effects of *N. fowleri*.

## DISCUSSION

One of the main impediments of *N. fowleri* management is the lack of understanding of its microbial ecology (Bartrand, Causey and Clancy 2014). While abiotic and biotic factors influence *N. fowleri* distribution and abundance, large knowledge gaps still exist, especially relating to *N. fowleri* within natural versus man-made environments. Specific areas of research needing investigation were identified within the descriptions of the current state of knowledge for each factor. Much of the previous research focused on either individually examining a few *N. fowleri* environmental factors in a controlled laboratory setting or examining a suite of selected factors from a small number of environments. These approaches, while valid, do not shed light on the potential interactive effects of multiple factors that likely occur in the environment. Thus, responses to multiple factors need to be assessed using both laboratory and environmental studies paired with viability assays and quantification of the amoebae. This requires that an efficient *N. fowleri* detection and quantification method is available, reducing time-intensive environmental cultivation approaches and permitting direct study to study comparisons. As evidenced by recent work conducted in DWDS, detection methods must also be able to assess viability of the amoebae and not just presence of nucleic acids.

With additional information on the distribution and abundance of *N. fowleri* in various environments, researchers will be able to determine which abiotic and biotic factors are most important for its growth and survival. Learning more about *N. fowleri* presence with other organisms may reveal potential sentinel species, inhibitory compounds or biological control agents that will provide needed information to make timely and informed water quality management decisions. Another area ripe for investigation is potential mechanisms for pathogen dispersal, as this topic has been largely unexplored but may be critical for understanding the distribution of *N. fowleri* in natural environments. Knowledge of the microbial ecology of *N. fowleri* is vital for water quality management strategies to prevent future PAM mortalities, especially as this disease is underreported (Matanock *et al*. 2018). As surface water temperatures continue to rise with climate change and environmental factors (e.g. pH, conductivity and water availability) are impacted as a result, PAM diagnoses will likely become more common (Martínez-Castillo *et al*. 2016; Maciver, Piñero and Lorenzo-Morales 2020) and methods to detect or mitigate the amoebae will be needed.
